# *rac*-2-Bromo-3-eth­oxy-1,3-bis­(4-methoxy­phen­yl)propan-1-one

**DOI:** 10.1107/S1600536808039238

**Published:** 2008-12-03

**Authors:** Hoong-Kun Fun, Samuel Robinson Jebas, Jyothi N. Rao, B. Kalluraya

**Affiliations:** a X-ray Crystallography Unit, School of Physics, Universiti Sains Malaysia, 11800 USM, Penang, Malaysia; b Department of Studies in Chemistry, Mangalore University, Mangalagangotri, Mangalore 574 199, India

## Abstract

In the racemic (*S,S*/*R,R*) title compound, C_19_H_21_BrO_4_, the two benzene rings are almost coplanar to each other, forming a dihedral angle of 3.58 (10)°. The crystal packing is strengthened by inter­molecular Br—O [2.9800 (16) Å] short contacts, which link the molecules into infinite one-dimensional chains along [001].

## Related literature

For the pharmacological applications of chalcones, see: Di Carlo *et al.* (1999 ▶); Dimmock *et al.* (1999 ▶); Go *et al.* (2005 ▶); Kalluraya *et al.* (1994 ▶); Rai *et al.* (2007 ▶). For bond-length data, see: Allen *et al.* (1987 ▶).
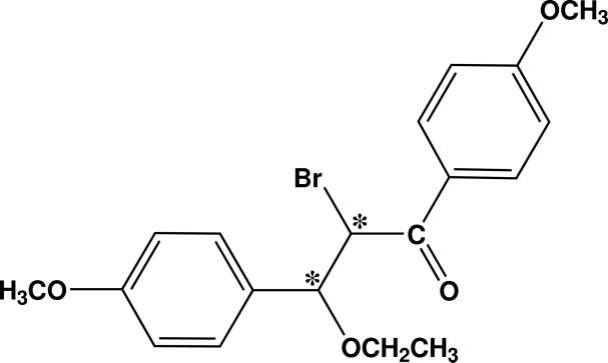


## Experimental

### 

#### Crystal data

C_19_H_21_BrO_4_
*M* *_r_* = 393.27
Monoclinic, 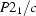

*a* = 12.2734 (10) Å
*b* = 15.3432 (12) Å
*c* = 10.4381 (8) Å
β = 114.399 (4)°
*V* = 1790.1 (3) Å^3^
*Z* = 4
Mo *K*α radiation
μ = 2.31 mm^−1^
*T* = 100.0 (1) K
0.55 × 0.38 × 0.19 mm

#### Data collection

Bruker SMART APEXII CCD area-detector diffractometer
Absorption correction: multi-scan (*SADABS*; Bruker, 2005 ▶) *T*_min_ = 0.351, *T*_max_ = 0.644
22919 measured reflections
5229 independent reflections
4345 reflections with *I* > 2σ(*I*)
*R*_int_ = 0.045

#### Refinement

*R*[*F* ^2^ > 2σ(*F* ^2^)] = 0.036
*wR*(*F* ^2^) = 0.103
*S* = 1.09
5229 reflections
219 parameters
H-atom parameters constrained
Δρ_max_ = 1.15 e Å^−3^
Δρ_min_ = −1.21 e Å^−3^

### 

Data collection: *APEX2* (Bruker, 2005 ▶); cell refinement: *SAINT* (Bruker, 2005 ▶); data reduction: *SAINT*; program(s) used to solve structure: *SHELXTL* (Sheldrick, 2008 ▶); program(s) used to refine structure: *SHELXTL*; molecular graphics: *SHELXTL*; software used to prepare material for publication: *SHELXTL* and *PLATON* (Spek, 2003 ▶).

## Supplementary Material

Crystal structure: contains datablocks global, I. DOI: 10.1107/S1600536808039238/dn2408sup1.cif

Structure factors: contains datablocks I. DOI: 10.1107/S1600536808039238/dn2408Isup2.hkl

Additional supplementary materials:  crystallographic information; 3D view; checkCIF report

## Figures and Tables

**Fig. 1.**
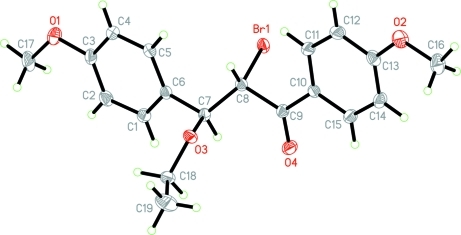
The molecular structure of the title compound, showing 50% probability displacement ellipsoids and the atom numbering scheme.

**Fig. 2.**
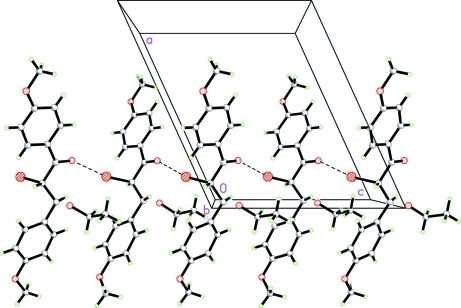
The crystal packing of the title compound, viewed down the b axis, showing the linking of the molecules by Br—O short contacts into an infinite one-dimensional chain along the [0 0 1]-direction.

## supplementary crystallographic information

### Comment

Chalcones, one of the major classes of natural products with widespread distribution in fruits, vegetables, spices, tea and soy based foodstuff, have recently been the subject of great interest for their interesting pharmacological activities (Di Carlo *et al.*, 1999). Chalcones and its derivatives have been reported to possess many useful properties including anti-inflammatory, antimicrobial, antifungal, antioxidant, cytotoxic, antitumor and anticancer activities (Dimmock *et al.*, 1999; Go *et al.*, 2005). Monobromo chalcones are used in the formation of many heterocyclic compounds having multiple applications (Kalluraya *et al.*, 1994; Rai *et al.*, 2007). Due to these varied applications, we have synthesized a new α-bromo chalcone and report its crystal structure.

Bond lengths and angles in (I) (Fig. 1) are found to have normal values (Allen *et al.*, 1987). There are two chiral centres C7 and C8 in the molecular structure therefore the centrosymmetic crystal is a racemate. The dihedral angle formed by the phenyl (C1—C6; C10—C15) rings is 3.58 (10)°, indicating that they are almost coplanar to each other.

The crystal packing is strengthened by intermolecular Br···O^i^=2.9800 (16)Å [symmetry code: *X*,1/2-Y,-1/2+*Z*] short contact. In the crystal packing, the molecules are linked into infinite one-dimensional chains along the [001] direction (Fig 2).

### Experimental

1,3-Di(*p*-anisyl)-2,3-dibromopropane (0.01 mol) when treated with ethanol(25 mL) at room temperature in presence of triethyl amine (0.02 mol) resulted in the formation of the title compound involving a nucleophilic substitution reaction.

### Refinement

H atoms were positioned geometrically (C—H=0.93–0.98 Å) and refined using a riding model with, *U*_iso_(H)=1.2U_equ_(C) and 1.5U_equ_(C_methyl_). A rotating group model was used for the methyl groups.

### Crystal data

**Table:**

C_19_H_21_BrO_4_	*F*(000) = 808
*M**_r_* = 393.27	*D*_x_ = 1.459 Mg m^−^^3^
Monoclinic, *P*2_1_/*c*	Mo *K*α radiation, λ = 0.71073 Å
Hall symbol: -P 2ybc	Cell parameters from 9847 reflections
*a* = 12.2734 (10) Å	θ = 2.3–35.9°
*b* = 15.3432 (12) Å	µ = 2.32 mm^−^^1^
*c* = 10.4381 (8) Å	*T* = 100 K
β = 114.399 (4)°	Block, colourless
*V* = 1790.1 (3) Å^3^	0.55 × 0.38 × 0.19 mm
*Z* = 4	

### Data collection

**Table:**

Bruker SMART APEXII CCD area-detector diffractometer	5229 independent reflections
Radiation source: fine-focus sealed tube	4345 reflections with *I* > 2σ(*I*)
graphite	*R*_int_ = 0.046
φ and ω scans	θ_max_ = 30.0°, θ_min_ = 1.8°
Absorption correction: multi-scan (*SADABS*; Bruker, 2005)	*h* = −17→17
*T*_min_ = 0.351, *T*_max_ = 0.644	*k* = −21→19
22919 measured reflections	*l* = −14→14

### Refinement

**Table:**

Refinement on *F*^2^	Primary atom site location: structure-invariant direct methods
Least-squares matrix: full	Secondary atom site location: difference Fourier map
*R*[*F*^2^ > 2σ(*F*^2^)] = 0.036	Hydrogen site location: inferred from neighbouring sites
*wR*(*F*^2^) = 0.103	H-atom parameters constrained
*S* = 1.09	*w* = 1/[σ^2^(*F*_o_^2^) + (0.0572*P*)^2^ + 0.6245*P*] where *P* = (*F*_o_^2^ + 2*F*_c_^2^)/3
5229 reflections	(Δ/σ)_max_ = 0.001
219 parameters	Δρ_max_ = 1.15 e Å^−^^3^
0 restraints	Δρ_min_ = −1.21 e Å^−^^3^

### Special details

**Table:**

Experimental. The data was collected with the Oxford Cyrosystem Cobra low-temperature attachment.
Geometry. All e.s.d.'s (except the e.s.d. in the dihedral angle between two l.s. planes) are estimated using the full covariance matrix. The cell e.s.d.'s are taken into account individually in the estimation of e.s.d.'s in distances, angles and torsion angles; correlations between e.s.d.'s in cell parameters are only used when they are defined by crystal symmetry. An approximate (isotropic) treatment of cell e.s.d.'s is used for estimating e.s.d.'s involving l.s. planes.
Refinement. Refinement of *F*^2^ against ALL reflections. The weighted *R*-factor *wR* and goodness of fit *S* are based on *F*^2^, conventional *R*-factors *R* are based on *F*, with *F* set to zero for negative *F*^2^. The threshold expression of *F*^2^ > σ(*F*^2^) is used only for calculating *R*-factors(gt) *etc*. and is not relevant to the choice of reflections for refinement. *R*-factors based on *F*^2^ are statistically about twice as large as those based on *F*, and *R*- factors based on ALL data will be even larger.

### Fractional atomic coordinates and isotropic or equivalent isotropic displacement parameters (Å^2^)

**Table:**

	*x*	*y*	*z*	*U*_iso_*/*U*_eq_	
Br1	0.141463 (16)	0.307078 (13)	0.897910 (19)	0.01800 (8)	
O1	−0.43693 (13)	0.28784 (11)	0.57932 (16)	0.0269 (3)	
O2	0.59815 (14)	0.61005 (11)	1.23343 (17)	0.0280 (3)	
O3	−0.01580 (12)	0.41258 (9)	1.14119 (14)	0.0178 (3)	
O4	0.23341 (13)	0.32415 (10)	1.25655 (16)	0.0242 (3)	
C1	−0.18733 (17)	0.26157 (13)	0.9281 (2)	0.0188 (4)	
H1A	−0.1636	0.2263	1.0075	0.023*	
C2	−0.29744 (18)	0.24568 (14)	0.8159 (2)	0.0215 (4)	
H2A	−0.3469	0.2013	0.8213	0.026*	
C3	−0.33187 (17)	0.29711 (13)	0.6964 (2)	0.0198 (4)	
C4	−0.25693 (17)	0.36403 (14)	0.6904 (2)	0.0196 (4)	
H4A	−0.2796	0.3980	0.6097	0.024*	
C5	−0.14917 (17)	0.38024 (13)	0.8036 (2)	0.0173 (4)	
H5A	−0.1010	0.4259	0.7992	0.021*	
C6	−0.11204 (17)	0.32845 (13)	0.9248 (2)	0.0156 (4)	
C7	0.00122 (16)	0.34564 (13)	1.05432 (19)	0.0153 (4)	
H7A	0.0254	0.2917	1.1093	0.018*	
C8	0.10736 (16)	0.38049 (13)	1.03024 (19)	0.0156 (4)	
H8A	0.0916	0.4404	0.9950	0.019*	
C9	0.22347 (17)	0.37698 (13)	1.16527 (19)	0.0168 (4)	
C10	0.32249 (16)	0.43701 (12)	1.17987 (19)	0.0156 (4)	
C11	0.31634 (18)	0.49576 (14)	1.0753 (2)	0.0207 (4)	
H11A	0.2487	0.4969	0.9906	0.025*	
C12	0.40986 (19)	0.55249 (15)	1.0964 (2)	0.0244 (4)	
H12A	0.4046	0.5917	1.0261	0.029*	
C13	0.51188 (18)	0.55101 (14)	1.2228 (2)	0.0214 (4)	
C14	0.51982 (18)	0.49233 (14)	1.3275 (2)	0.0215 (4)	
H14A	0.5877	0.4910	1.4119	0.026*	
C15	0.42559 (17)	0.43567 (13)	1.3052 (2)	0.0194 (4)	
H15A	0.4313	0.3961	1.3751	0.023*	
C16	0.7050 (2)	0.61116 (17)	1.3607 (3)	0.0305 (5)	
H16A	0.7593	0.6538	1.3532	0.046*	
H16B	0.7420	0.5547	1.3762	0.046*	
H16C	0.6855	0.6257	1.4382	0.046*	
C17	−0.52119 (18)	0.22532 (17)	0.5863 (2)	0.0286 (5)	
H17A	−0.5905	0.2245	0.4983	0.043*	
H17B	−0.5444	0.2410	0.6605	0.043*	
H17C	−0.4850	0.1686	0.6046	0.043*	
C18	−0.07984 (19)	0.38291 (14)	1.2207 (2)	0.0210 (4)	
H18A	−0.0499	0.3265	1.2623	0.025*	
H18B	−0.1643	0.3773	1.1602	0.025*	
C19	−0.0613 (3)	0.44917 (18)	1.3337 (3)	0.0375 (6)	
H19A	−0.1025	0.4308	1.3898	0.056*	
H19B	−0.0923	0.5045	1.2913	0.056*	
H19C	0.0226	0.4546	1.3923	0.056*	

### Atomic displacement parameters (Å^2^)

**Table:**

	*U*^11^	*U*^22^	*U*^33^	*U*^12^	*U*^13^	*U*^23^
Br1	0.01397 (11)	0.02230 (13)	0.01772 (11)	0.00063 (7)	0.00655 (8)	−0.00428 (7)
O1	0.0155 (7)	0.0366 (9)	0.0219 (8)	−0.0060 (6)	0.0008 (6)	−0.0019 (6)
O2	0.0209 (7)	0.0318 (9)	0.0297 (8)	−0.0109 (7)	0.0088 (6)	−0.0012 (7)
O3	0.0188 (6)	0.0194 (7)	0.0179 (6)	−0.0001 (5)	0.0103 (5)	−0.0021 (5)
O4	0.0190 (7)	0.0292 (8)	0.0205 (7)	−0.0047 (6)	0.0043 (6)	0.0068 (6)
C1	0.0180 (9)	0.0186 (10)	0.0199 (9)	−0.0004 (7)	0.0080 (7)	0.0015 (7)
C2	0.0171 (9)	0.0239 (11)	0.0244 (10)	−0.0048 (8)	0.0096 (8)	−0.0017 (8)
C3	0.0128 (8)	0.0250 (11)	0.0199 (9)	−0.0007 (7)	0.0050 (7)	−0.0041 (7)
C4	0.0169 (8)	0.0239 (10)	0.0172 (9)	0.0014 (8)	0.0061 (7)	0.0017 (7)
C5	0.0156 (8)	0.0177 (9)	0.0200 (9)	−0.0007 (7)	0.0089 (7)	−0.0003 (7)
C6	0.0136 (8)	0.0174 (9)	0.0162 (8)	0.0007 (7)	0.0066 (7)	−0.0019 (7)
C7	0.0139 (8)	0.0160 (9)	0.0159 (8)	0.0002 (7)	0.0060 (7)	−0.0007 (7)
C8	0.0141 (8)	0.0180 (9)	0.0150 (8)	0.0006 (7)	0.0062 (7)	−0.0017 (7)
C9	0.0144 (8)	0.0195 (10)	0.0152 (8)	−0.0004 (7)	0.0048 (7)	−0.0014 (7)
C10	0.0141 (8)	0.0175 (9)	0.0160 (8)	−0.0008 (7)	0.0069 (7)	−0.0009 (7)
C11	0.0168 (9)	0.0266 (11)	0.0166 (9)	−0.0029 (8)	0.0050 (7)	0.0020 (7)
C12	0.0217 (10)	0.0305 (12)	0.0212 (10)	−0.0057 (9)	0.0090 (8)	0.0037 (8)
C13	0.0175 (9)	0.0241 (11)	0.0239 (10)	−0.0060 (8)	0.0097 (8)	−0.0050 (8)
C14	0.0164 (9)	0.0251 (11)	0.0183 (9)	−0.0022 (8)	0.0024 (7)	−0.0016 (7)
C15	0.0181 (9)	0.0211 (10)	0.0168 (9)	−0.0009 (8)	0.0050 (7)	0.0012 (7)
C16	0.0180 (10)	0.0336 (13)	0.0356 (12)	−0.0075 (9)	0.0067 (9)	−0.0066 (10)
C17	0.0144 (9)	0.0372 (13)	0.0320 (12)	−0.0058 (9)	0.0075 (9)	−0.0100 (10)
C18	0.0234 (10)	0.0237 (10)	0.0197 (9)	0.0043 (8)	0.0126 (8)	0.0038 (7)
C19	0.0482 (15)	0.0422 (15)	0.0324 (13)	−0.0034 (12)	0.0272 (12)	−0.0100 (11)

### Geometric parameters (Å, °)

**Table:**

Br1—C8	1.9569 (18)	C9—C10	1.482 (3)
O1—C3	1.369 (2)	C10—C11	1.393 (3)
O1—C17	1.434 (3)	C10—C15	1.395 (3)
O2—C13	1.363 (2)	C11—C12	1.384 (3)
O2—C16	1.431 (3)	C11—H11A	0.9300
O3—C18	1.433 (2)	C12—C13	1.394 (3)
O3—C7	1.441 (2)	C12—H12A	0.9300
O4—C9	1.218 (2)	C13—C14	1.388 (3)
C1—C6	1.391 (3)	C14—C15	1.388 (3)
C1—C2	1.396 (3)	C14—H14A	0.9300
C1—H1A	0.9300	C15—H15A	0.9300
C2—C3	1.387 (3)	C16—H16A	0.9600
C2—H2A	0.9300	C16—H16B	0.9600
C3—C4	1.397 (3)	C16—H16C	0.9600
C4—C5	1.384 (3)	C17—H17A	0.9600
C4—H4A	0.9300	C17—H17B	0.9600
C5—C6	1.401 (3)	C17—H17C	0.9600
C5—H5A	0.9300	C18—C19	1.502 (3)
C6—C7	1.509 (3)	C18—H18A	0.9700
C7—C8	1.522 (3)	C18—H18B	0.9700
C7—H7A	0.9800	C19—H19A	0.9600
C8—C9	1.536 (3)	C19—H19B	0.9600
C8—H8A	0.9800	C19—H19C	0.9600
			
Br1···O4^i^	2.9800 (16)		
			
C3—O1—C17	117.21 (17)	C12—C11—C10	120.63 (19)
C13—O2—C16	117.85 (18)	C12—C11—H11A	119.7
C18—O3—C7	113.25 (15)	C10—C11—H11A	119.7
C6—C1—C2	122.07 (18)	C11—C12—C13	120.13 (19)
C6—C1—H1A	119.0	C11—C12—H12A	119.9
C2—C1—H1A	119.0	C13—C12—H12A	119.9
C3—C2—C1	119.00 (19)	O2—C13—C14	124.58 (19)
C3—C2—H2A	120.5	O2—C13—C12	115.48 (19)
C1—C2—H2A	120.5	C14—C13—C12	119.94 (19)
O1—C3—C2	124.66 (19)	C15—C14—C13	119.47 (18)
O1—C3—C4	115.54 (18)	C15—C14—H14A	120.3
C2—C3—C4	119.81 (18)	C13—C14—H14A	120.3
C5—C4—C3	120.56 (18)	C14—C15—C10	121.22 (18)
C5—C4—H4A	119.7	C14—C15—H15A	119.4
C3—C4—H4A	119.7	C10—C15—H15A	119.4
C4—C5—C6	120.57 (18)	O2—C16—H16A	109.5
C4—C5—H5A	119.7	O2—C16—H16B	109.5
C6—C5—H5A	119.7	H16A—C16—H16B	109.5
C1—C6—C5	117.97 (17)	O2—C16—H16C	109.5
C1—C6—C7	118.85 (17)	H16A—C16—H16C	109.5
C5—C6—C7	123.05 (17)	H16B—C16—H16C	109.5
O3—C7—C6	111.65 (15)	O1—C17—H17A	109.5
O3—C7—C8	102.12 (14)	O1—C17—H17B	109.5
C6—C7—C8	116.62 (15)	H17A—C17—H17B	109.5
O3—C7—H7A	108.7	O1—C17—H17C	109.5
C6—C7—H7A	108.7	H17A—C17—H17C	109.5
C8—C7—H7A	108.7	H17B—C17—H17C	109.5
C7—C8—C9	111.65 (15)	O3—C18—C19	107.37 (18)
C7—C8—Br1	111.20 (13)	O3—C18—H18A	110.2
C9—C8—Br1	103.86 (12)	C19—C18—H18A	110.2
C7—C8—H8A	110.0	O3—C18—H18B	110.2
C9—C8—H8A	110.0	C19—C18—H18B	110.2
Br1—C8—H8A	110.0	H18A—C18—H18B	108.5
O4—C9—C10	121.35 (17)	C18—C19—H19A	109.5
O4—C9—C8	119.74 (18)	C18—C19—H19B	109.5
C10—C9—C8	118.89 (16)	H19A—C19—H19B	109.5
C11—C10—C15	118.61 (18)	C18—C19—H19C	109.5
C11—C10—C9	123.01 (17)	H19A—C19—H19C	109.5
C15—C10—C9	118.36 (17)	H19B—C19—H19C	109.5
			
C6—C1—C2—C3	1.2 (3)	C7—C8—C9—O4	25.2 (3)
C17—O1—C3—C2	5.5 (3)	Br1—C8—C9—O4	−94.68 (19)
C17—O1—C3—C4	−174.36 (19)	C7—C8—C9—C10	−156.22 (17)
C1—C2—C3—O1	179.59 (19)	Br1—C8—C9—C10	83.87 (18)
C1—C2—C3—C4	−0.5 (3)	O4—C9—C10—C11	177.2 (2)
O1—C3—C4—C5	179.04 (18)	C8—C9—C10—C11	−1.3 (3)
C2—C3—C4—C5	−0.9 (3)	O4—C9—C10—C15	−4.0 (3)
C3—C4—C5—C6	1.6 (3)	C8—C9—C10—C15	177.47 (17)
C2—C1—C6—C5	−0.5 (3)	C15—C10—C11—C12	−1.0 (3)
C2—C1—C6—C7	175.54 (18)	C9—C10—C11—C12	177.7 (2)
C4—C5—C6—C1	−0.9 (3)	C10—C11—C12—C13	0.4 (3)
C4—C5—C6—C7	−176.77 (18)	C16—O2—C13—C14	0.7 (3)
C18—O3—C7—C6	76.10 (19)	C16—O2—C13—C12	−179.8 (2)
C18—O3—C7—C8	−158.56 (15)	C11—C12—C13—O2	−179.4 (2)
C1—C6—C7—O3	−94.3 (2)	C11—C12—C13—C14	0.2 (3)
C5—C6—C7—O3	81.5 (2)	O2—C13—C14—C15	179.4 (2)
C1—C6—C7—C8	148.80 (18)	C12—C13—C14—C15	−0.1 (3)
C5—C6—C7—C8	−35.3 (3)	C13—C14—C15—C10	−0.5 (3)
O3—C7—C8—C9	70.12 (18)	C11—C10—C15—C14	1.1 (3)
C6—C7—C8—C9	−167.90 (16)	C9—C10—C15—C14	−177.72 (19)
O3—C7—C8—Br1	−174.39 (11)	C7—O3—C18—C19	164.38 (17)
C6—C7—C8—Br1	−52.41 (19)		

Symmetry codes:  (i) *x*, −*y*+1/2, *z*−1/2.
